# Patterns of etanercept use in juvenile idiopathic arthritis in the Childhood Arthritis and Rheumatology Research Alliance Registry

**DOI:** 10.1186/s12969-021-00625-y

**Published:** 2021-08-21

**Authors:** Timothy Beukelman, Aimee Lougee, Roland A. Matsouaka, David Collier, Dax G. Rumsey, Jennifer Schenfeld, Scott Stryker, Marinka Twilt, Yukiko Kimura, for the CARRA Registry Investigators, N. Abel, K. Abulaban, A. Adams, M. Adams, R. Agbayani, J. Aiello, S. Akoghlanian, C. Alejandro, E. Allenspach, R. Alperin, M. Alpizar, G. Amarilyo, W. Ambler, E. Anderson, S. Ardoin, S. Armendariz, E. Baker, I. Balboni, S. Balevic, L. Ballenger, S. Ballinger, N. Balmuri, F. Barbar-Smiley, L. Barillas-Arias, M. Basiaga, K. Baszis, M. Becker, H. Bell-Brunson, E. Beltz, H. Benham, S. Benseler, W. Bernal, T. Beukelman, T. Bigley, B. Binstadt, C. Black, M. Blakley, J. Bohnsack, J. Boland, A. Boneparth, S. Bowman, C. Bracaglia, E. Brooks, M. Brothers, A. Brown, H. Brunner, M. Buckley, M. Buckley, H. Bukulmez, D. Bullock, B. Cameron, S. Canna, L. Cannon, P. Carper, V. Cartwright, E. Cassidy, L. Cerracchio, E. Chalom, J. Chang, A. Chang-Hoftman, V. Chauhan, P. Chira, T. Chinn, K. Chundru, H. Clairman, D. Co, A. Confair, H. Conlon, R. Connor, A. Cooper, J. Cooper, S. Cooper, C. Correll, R. Corvalan, D. Costanzo, R. Cron, L. Curiel-Duran, T. Curington, M. Curry, A. Dalrymple, A. Davis, C. Davis, C. Davis, T. Davis, F. De Benedetti, D. De Ranieri, J. Dean, F. Dedeoglu, M. DeGuzman, N. Delnay, V. Dempsey, E. DeSantis, T. Dickson, J. Dingle, B. Donaldson, E. Dorsey, S. Dover, J. Dowling, J. Drew, K. Driest, Q. Du, K. Duarte, D. Durkee, E. Duverger, J. Dvergsten, A. Eberhard, M. Eckert, K. Ede, B. Edelheit, C. Edens, C. Edens, Y. Edgerly, M. Elder, B. Ervin, S. Fadrhonc, C. Failing, D. Fair, M. Falcon, L. Favier, S. Federici, B. Feldman, J. Fennell, I. Ferguson, P. Ferguson, B. Ferreira, R. Ferrucho, K. Fields, T. Finkel, M. Fitzgerald, C. Fleming, O. Flynn, L. Fogel, E. Fox, M. Fox, L. Franco, M. Freeman, K. Fritz, S. Froese, R. Fuhlbrigge, J. Fuller, N. George, K. Gerhold, D. Gerstbacher, M. Gilbert, M. Gillispie-Taylor, E. Giverc, C. Godiwala, I. Goh, H. Goheer, D. Goldsmith, E. Gotschlich, A. Gotte, B. Gottlieb, C. Gracia, T. Graham, S. Grevich, T. Griffin, J. Griswold, A. Grom, M. Guevara, P. Guittar, M. Guzman, M. Hager, T. Hahn, O. Halyabar, E. Hammelev, M. Hance, A. Hanson, L. Harel, S. Haro, J. Harris, O. Harry, E. Hartigan, J. Hausmann, A. Hay, K. Hayward, J. Heiart, K. Hekl, L. Henderson, M. Henrickson, A. Hersh, K. Hickey, P. Hill, S. Hillyer, L. Hiraki, M. Hiskey, P. Hobday, C. Hoffart, M. Holland, M. Hollander, S. Hong, M. Horwitz, J. Hsu, A. Huber, J. Huggins, J. Hui-Yuen, C. Hung, J. Huntington, A. Huttenlocher, M. Ibarra, L. Imundo, C. Inman, A. Insalaco, A. Jackson, S. Jackson, K. James, G. Janow, J. Jaquith, S. Jared, N. Johnson, J. Jones, J. Jones, J. Jones, K. Jones, S. Jones, S. Joshi, L. Jung, C. Justice, A. Justiniano, N. Karan, K. Kaufman, A. Kemp, E. Kessler, U. Khalsa, B. Kienzle, S. Kim, Y. Kimura, D. Kingsbury, M. Kitcharoensakkul, T. Klausmeier, K. Klein, M. Klein-Gitelman, B. Kompelien, A. Kosikowski, L. Kovalick, J. Kracker, S. Kramer, C. Kremer, J. Lai, J. Lam, B. Lang, S. Lapidus, B. Lapin, A. Lasky, D. Latham, E. Lawson, R. Laxer, P. Lee, P. Lee, T. Lee, L. Lentini, M. Lerman, D. Levy, S. Li, S. Lieberman, L. Lim, C. Lin, N. Ling, M. Lingis, M. Lo, D. Lovell, D. Lowman, N. Luca, S. Lvovich, C. Madison, J. Madison, S. Magni Manzoni, B. Malla, J. Maller, M. Malloy, M. Mannion, C. Manos, L. Marques, A. Martyniuk, T. Mason, S. Mathus, L. McAllister, K. McCarthy, K. McConnell, E. McCormick, D. McCurdy, P. Mc Curdy Stokes, S. McGuire, I. McHale, A. McMonagle, C. McMullen-Jackson, E. Meidan, E. Mellins, E. Mendoza, R. Mercado, A. Merritt, L. Michalowski, P. Miettunen, M. Miller, D. Milojevic, E. Mirizio, E. Misajon, M. Mitchell, R. Modica, S. Mohan, K. Moore, L. Moorthy, S. Morgan, E. Morgan Dewitt, C. Moss, T. Moussa, V. Mruk, A. Murphy, E. Muscal, R. Nadler, B. Nahal, K. Nanda, N. Nasah, L. Nassi, S. Nativ, M. Natter, J. Neely, B. Nelson, L. Newhall, L. Ng, J. Nicholas, R. Nicolai, P. Nigrovic, J. Nocton, B. Nolan, E. Oberle, B. Obispo, B. O’Brien, T. O’Brien, O. Okeke, M. Oliver, J. Olson, K. O’Neil, K. Onel, A. Orandi, M. Orlando, S. Osei-Onomah, R. Oz, E. Pagano, A. Paller, N. Pan, S. Panupattanapong, M. Pardeo, J. Paredes, A. Parsons, J. Patel, K. Pentakota, P. Pepmueller, T. Pfeiffer, K. Phillippi, D. Pires Marafon, K. Phillippi, L. Ponder, R. Pooni, S. Prahalad, S. Pratt, S. Protopapas, B. Puplava, J. Quach, M. Quinlan-Waters, C. Rabinovich, S. Radhakrishna, J. Rafko, J. Raisian, A. Rakestraw, C. Ramirez, E. Ramsay, S. Ramsey, R. Randell, A. Reed, A. Reed, A. Reed, H. Reid, K. Remmel, A. Repp, A. Reyes, A. Richmond, M. Riebschleger, S. Ringold, M. Riordan, M. Riskalla, M. Ritter, R. Rivas-Chacon, A. Robinson, E. Rodela, M. Rodriquez, K. Rojas, T. Ronis, M. Rosenkranz, B. Rosolowski, H. Rothermel, D. Rothman, E. Roth-Wojcicki, K. Rouster-Stevens, T. Rubinstein, N. Ruth, N. Saad, S. Sabbagh, E. Sacco, R. Sadun, C. Sandborg, A. Sanni, L. Santiago, A. Sarkissian, S. Savani, L. Scalzi, L. Schanberg, S. Scharnhorst, K. Schikler, A. Schlefman, H. Schmeling, K. Schmidt, E. Schmitt, R. Schneider, K. Schollaert-Fitch, G. Schulert, T. Seay, C. Seper, J. Shalen, R. Sheets, A. Shelly, S. Shenoi, K. Shergill, J. Shirley, M. Shishov, C. Shivers, E. Silverman, N. Singer, V. Sivaraman, J. Sletten, A. Smith, C. Smith, J. Smith, J. Smith, E. Smitherman, J. Soep, M. Son, S. Spence, L. Spiegel, J. Spitznagle, R. Sran, H. Srinivasalu, H. Stapp, K. Steigerwald, Y. Sterba Rakovchik, S. Stern, A. Stevens, B. Stevens, R. Stevenson, K. Stewart, C. Stingl, J. Stokes, M. Stoll, E. Stringer, S. Sule, J. Sumner, R. Sundel, M. Sutter, R. Syed, G. Syverson, A. Szymanski, S. Taber, R. Tal, A. Tambralli, A. Taneja, T. Tanner, S. Tapani, G. Tarshish, S. Tarvin, L. Tate, A. Taxter, J. Taylor, M. Terry, M. Tesher, A. Thatayatikom, B. Thomas, K. Tiffany, T. Ting, A. Tipp, D. Toib, K. Torok, C. Toruner, H. Tory, M. Toth, S. Tse, V. Tubwell, M. Twilt, S. Uriguen, T. Valcarcel, H. Van Mater, L. Vannoy, C. Varghese, N. Vasquez, K. Vazzana, R. Vehe, K. Veiga, J. Velez, J. Verbsky, G. Vilar, N. Volpe, E. von Scheven, S. Vora, J. Wagner, L. Wagner-Weiner, D. Wahezi, H. Waite, J. Walker, H. Walters, T. Wampler Muskardin, L. Waqar, M. Waterfield, M. Watson, A. Watts, P. Weiser, J. Weiss, P. Weiss, E. Wershba, A. White, C. Williams, A. Wise, J. Woo, L. Woolnough, T. Wright, E. Wu, A. Yalcindag, M. Yee, E. Yen, R. Yeung, K. Yomogida, Q. Yu, R. Zapata, A. Zartoshti, A. Zeft, R. Zeft, Y. Zhang, Y. Zhao, A. Zhu, C. Zic

**Affiliations:** 1 Department of Pediatrics, University of Alabama at Birmingham, CPPN G10, 1600 7th Ave South, Birmingham, AL 35233 USA; 2 Duke University, Duke Clinical Research Institute, 200 Morris Street, Durham, NC 27701 USA; 3 Department of Biostatistics and Bioinformatics, Duke University, Duke Clinical Research Institute, 200 Morris Street, Durham, NC 27701 USA; 4 Global Medical Affairs, Amgen Inc., One Amgen Center Drive, Thousand Oaks, California 91320-1799 USA; 5 Department of Pediatrics, University of Alberta, 3-502 ECHA; 11405 87 Ave NW, Edmonton, Alberta T6G 1C9 Canada; 6 Center for Observational Research, Amgen Inc., One Amgen Center Drive, Thousand Oaks, California 91320-1799 USA; 7 Department of Pediatrics, Alberta Children’s Hospital, Cumming School of Medicine, University of Calgary, 28 Oki Drive NW, Calgary, Alberta T3B 6A8 Canada; 8 Joseph M. Sanzari Children’s Hospital, Hackensack University Medical Center, Hackensack Meridian School of Medicine, Hackensack, NJ USA

**Keywords:** Arthritis, juvenile, Cohort studies, Etanercept, Anti-TNF, Paediatric rheumatology, Registry

## Abstract

**Background:**

We aimed to characterize etanercept (ETN) use in juvenile idiopathic arthritis (JIA) patients enrolled in the Childhood Arthritis and Rheumatology Research Alliance (CARRA) Registry.

**Methods:**

The CARRA Registry is a convenience cohort of patients with paediatric onset rheumatic diseases, including JIA. JIA patients treated with ETN for whom the month and year of ETN initiation were available were included. Patterns of ETN and methotrexate (MTX) use were categorized as follows: combination therapy (ETN and MTX started concurrently), step-up therapy (MTX started first and ETN added later), switchers (MTX started and then stopped when or before ETN started), MTX add-on (ETN started first and MTX added later), and ETN only (no MTX use). Data were described using parametric and non-parametric statistics as appropriate.

**Results:**

Two thousand thirty-two of the five thousand six hundred forty-one patients with JIA met inclusion criteria (74% female, median age at diagnosis 6.0 years [interquartile range 2.0, 11.0]. Most patients (66.9%) were treated with a non-biologic disease modifying anti-rheumatic drug (DMARD), primarily MTX, prior to ETN. There was significant variability in patterns of MTX use prior to starting ETN. Step-up therapy was the most common approach. Only 34.0% of persistent oligoarticular JIA patients continued treatment with a non-biologic DMARD 3 months or more after ETN initiation. ETN persistence overall was 66.3, 49.4, and 37.3% at 24, 36 and 48 months respectively. ETN persistence among spondyloarthritis patients (enthesitis related arthritis and psoriatic JIA) varied by MTX initiation pattern, with higher ETN persistence rates in those who initiated combination therapy (68.9%) and switchers/ETN only (73.3%) patients compared to step-up (65.4%) and MTX add-on (51.1%) therapy.

**Conclusion:**

This study characterizes contemporary patterns of ETN use in the CARRA Registry. Treatment was largely in keeping with American College of Rheumatology guidelines.

**Supplementary Information:**

The online version contains supplementary material available at 10.1186/s12969-021-00625-y.

## Background

Juvenile idiopathic arthritis (JIA) is the most common paediatric rheumatic disease with prevalence rates between 0.038 and 4 per 1000 [1], and encompasses 7 categories of inflammatory arthritis [2]. Prior to the advent of biologics, long-term damage and disability were common. In 1999, etanercept (ETN) became the first anti-tumor necrosis factor (anti-TNF) therapy approved by the Food and Drug Administration (FDA) for polyarticular JIA, and has since been shown to be effective in multiple categories of JIA (reviewed in [3–8]). The American College of Rheumatology (ACR) includes anti-TNF therapy in its JIA treatment guidelines [9, 10]. Despite its longstanding availability, ETN’s clinical use in North America in JIA has not been well described. Additionally, ETN is often prescribed in combination therapy with methotrexate (MTX), but the frequency and patterns of combination therapy have not been well characterized.

The Childhood Arthritis and Rheumatology Research Alliance (CARRA) Registry is a large, multi-centre convenience cohort of paediatric patients with a variety of rheumatic diseases, including JIA. This study describes ETN use and patterns of concurrent MTX therapy in patients with JIA enrolled in the CARRA Registry.

## Methods

### Data source

The CARRA Registry is a disease based registry of a convenience cohort of paediatric patients with rheumatic diseases including JIA [11]. Initial data collection focused on patients within 6 months of JIA diagnosis or newly starting methotrexate or a biologic, and shortly thereafter expanded to include patients with a history of ≥5 joints or systemic JIA (SJIA). In 2017, it was expanded to all JIA patients. Patients up to 21 years of age are enrolled at paediatric rheumatology centers. Retrospective data is collected at enrolment, and then prospective data is collected approximately every 6 months, as well as whenever a biologic or non-biologic DMARD for JIA is initiated. Data collected include date of diagnosis, clinician-reported International League Against Rheumatism (ILAR) JIA category, medications, laboratory data, clinical features within the 2 weeks preceding each study visit, the Childhood Heath Assessment Questionnaire (CHAQ), which is a validated measure of functional status, the Physician Global Assessment of disease activity, the Patient/Parent Global Assessment of well-being, and several Patient-Reported Outcome Measurement Information System (PROMIS) measures [12]. The Clinical Juvenile Arthritis Disease Activity Score 10 (cJADAS10), a validated measure of disease activity [13], is calculated for each study visit. The physician and patient/parent global assessments are included in the American College of Rheumatology core set of measures of disease activity [14], and are components of the cJADAS10 [13]. Pain is assessed using an 11-point numerical rating scale from 0 to 10. Data collected from June 30, 2015 to June 30, 2018 from 60 U.S. and 3 Canadian clinical sites were included in this analysis. Informed consent was obtained for participation in the CARRA Registry, and data collection complies with the Declaration of Helsinki. The CARRA Registry is approved by the Duke University IRB (Pro00054616),

### Patient population

In order to gain a broad understanding of factors leading to ETN initiation for JIA and the interplay between methotrexate and ETN in real-world settings, all JIA patients in the CARRA Registry ever treated with ETN were included, except for patients with a concurrent diagnosis of a secondary rheumatologic condition or inflammatory bowel disease prior to ETN initiation. Patients missing year of diagnosis were excluded from some of the analyses. There were three patient cohorts: (1) All eligible patients; (2) Patients with at least one visit at least 6 months after beginning treatment with ETN, irrespective of whether ETN was started before or after enrolment in the Registry, to assess patterns of MTX use near the time of ETN initiation (MTX Assessment Cohort); (3) Participants with a study visit within 30 days before or after their ETN start date to assess clinical status near the time of ETN initiation (ETN Initiator Cohort).

The physician assigned JIA category closest to ETN initiation was used where available, while patients who had been treated with ETN prior to Registry enrolment were classified according to their JIA category at the enrolment visit. MTX and ETN were considered to have been initiated concurrently if started within 30 days of each other, to allow for delays due to medication coverage approvals and insurance requirements. The addition of ETN after more than 1 month of MTX treatment with continuation of MTX for at least 1 month after starting ETN was classified as step-up therapy. Clinical characteristics of patients grouped according to their pattern of MTX and ETN use were described. Patients who started ETN within 3 months of diagnosis were compared to those who started ETN more than 3 months after diagnosis to highlight characteristics associated with early ETN use. Non-biologic DMARD discontinuation within 3 months of ETN initiation versus 3 or more months after ETN initiation was also assessed in the ETN Initiator Cohort. The 3-month timeline was chosen to allow adequate time for a complete response to ETN prior to discontinuation of the non-biologic DMARD.

### Statistical analysis

Descriptive statistics including means, standard deviations, medians, interquartile ranges (IQR) for continuous variables and proportions for categorical variables were calculated as appropriate. *P*-values for categorical variables were calculated when at least 50% of the cell counts had more than zero records. Tests for difference between groups were performed between categorical variables using Pearson’s chi-square or Fisher’s exact tests, depending on expected cell counts. Statistical comparisons for continuous measures were conducted using Wilcoxon rank sum, Kruskal-Wallis, analysis of variance (ANOVA), or t-tests depending on normality and the number of groups being compared. P-values for comparisons of medians were calculated using a Wilcoxon rank-sum test of medians. Where appropriate, *P*-value significance levels were adjusted for multiple comparisons by multiplying by a factor of N choose n, depending on the number of categories being compared and the total number of categories possible. Kaplan Meier (KM) estimates were calculated to describe ETN persistence by JIA category, pattern of MTX use around the time of ETN initiation, and pattern of MTX use the time of ETN initiation in polyarticular JIA (combining rheumatoid factor (RF) positive and negative polyarticular JIA (pJIA) patients), and in spondyloarthritides (enthesitis related arthritis (ERA) and psoriatic JIA (PsJIA) combined). Persistence was assessed for the first course of ETN only, with gaps of > 90 days considered to be a new medication course. Medication discontinuation for any reason was included in the analyses. Medication records with missing start dates (i.e., only month and year recorded) were imputed with 15th of the month. Case report form data was collected via Medidata Rave, and all analyses were performed using SAS v 9.4 (Cary, NC).

## Results

At the time of data extraction, 2032 of the 5641 JIA patients in the Registry met inclusion criteria. In this cohort of all eligible patients, 73.6% were female and 77.2% were white. ILAR categories were: 22.5% oligoarthritis (OligoJIA), 42.6% RF- polyarticular JIA (RF-pJIA), 11.9% RF+ polyarticular JIA (RF + pJIA), 8.1% PsJIA, 10.2% ERA, 2.6% systemic JIA (SJIA), and 2.0% undifferentiated arthritis. Overall, 66.9% of the 2032 patients were treated with at least 1 non-biologic DMARD at least 1 month prior to ETN initiation; in 96.8% of cases this DMARD was MTX. Subcutaneous MTX use only was most common (43.5% of MTX users), followed by oral use only (24.6% of MTX users), with the remainder having been prescribed both forms of MTX or intramuscular MTX. In 94.5% ETN was the first biologic prescribed and was started a median of 3.7 months (IQR 1.1, 10.4) after the first non-biologic DMARD, and 5.8 months (IQR 2.1, 21.8) after diagnosis.

Table 1 shows patterns of MTX use in individuals with ≥1 visit 6 months after starting ETN (*n* = 1681, MTX Assessment Cohort). In all categories of JIA, the most common treatment approach was the addition of ETN after initiating MTX (step-up therapy). In contrast to this step-up therapy approach, 5.1% of this cohort discontinued MTX within 1 month of starting ETN, and another 5.1% discontinued MTX more than 1 month before starting ETN. ERA patients were more likely than any other ILAR category to use ETN without methotrexate. Interestingly, MTX was added to ETN therapy at least 1 month after ETN initiation in 8.6% of patients (MTX add-on), most commonly in SJIA, though the number of SJIA patients was small. A similar percentage of patients with RF + pJIA and PsJIA started MTX and ETN concurrently (combination therapy), as did OligoJIA patients for whom JIA subcategory (i.e., persistent versus extended) was unknown, though the number of the latter was small.

**Table 1:** Patterns of methotrexate (MTX) use at the start of etanercept therapy^a^ (MTX Assessment Cohort, *n* = 1681)

Characteristic	Combination therapy^b^	Step-up therapy^c^	ETN proximate switchers^d^	ETN remote switchers^e^	MTX add-on^f^	ETN only
All patients (n = 1681), n (%)	258 (15.3)	892 (53.1)	86 (5.1)	86 (5.1)	144 (8.6)	215 (12.8)
JIA^g^ category, n (%)^h^						
Oligoarthritis (*n* = 366)	34 (9.3)	184 (50.3)	34 (9.3)	32 (8.7)	29 (7.9)	53 (14.5)
Persistent (*n* = 159)	13 (8.2)	74 (46.5)	11 (6.9)	13 (8.2)	12 (7.5)	36 (22.6)
Extended (*n* = 178)	15 (8.4)	99 (55.6)	18 (10.1)	19 (10.7)	15 (8.4)	12 (6.7)
Unknown (*n* = 29)	6 (20.7)	11 (37.9)	5 (17.2)	0	2 (6.9)	5 (17.2)
Polyarthritis (RF-)^i^ (*n* = 736)	113 (15.4)	437 (59.4)	36 (4.9)	31 (4.2)	57 (7.7)	62 (8.4)
Polyarthritis (RF+) (*n* = 208)	49 (23.6)	109 (52.4)	3 (1.4)	7 (3.4)	23 (11.1)	17 (8.2)
Psoriatic arthritis (*n* = 134)	29 (21.6)	59 (44.0)	4 (3.0)	5 (3.7)	12 (9.0)	25 (18.7)
Enthesitis related arthritis (*n* = 163)	23 (14.1)	65 (39.9)	7 (4.3)	4 (2.5)	13 (8.0)	51 (31.3)
Systemic arthritis (*n* = 40)	4 (10.0)	23 (57.5)	1 (2.5)	2 (5.0)	9 (22.5)	1 (2.5)
Undifferentiated arthritis (*n* = 34)	6 (17.6)	15 (44.1)	1 (2.9)	5 (14.7)	1 (2.9)	6 (17.6)

^a^ In patients with at least 1 study visit 6 months after starting etanercept; ^b^ Combination therapy = methotrexate (MTX) started concurrently with etanercept (ETN); ^c^ step-up therapy = MTX started > 1 month prior to ETN and continued > 1 month after ETN initiation; ^d^ proximate switchers = MTX started> 1 month prior to ETN and discontinued within 1 month prior to or after ETN; ^e^ ETN remote switchers = MTX discontinued > 1 month prior to start of ETN; ^f^ MTX add-on = MTX started > 1 month after starting ETN; ^g^*JIA* juvenile idiopathic arthritis; ^h^ Denominator for percentage calculations is the n for that category of JIA; ^i^*RF* rheumatoid factor

Within the ETN Initiator Cohort (those with clinical data within 30 days of starting ETN), 40.3% started ETN ≤3 months after diagnosis, while 59.7% started ETN > 3 months after diagnosis. Patients who started ETN within 3 months of JIA diagnosis had a higher median joint count; 8.0 (IQR 4.0, 16.0) vs 3.0 (IQR 1.0, 7.0), *p* < 0.001), higher cJADAS10 (median 17.0 (IQR 11.0, 22.0) vs 11.0 (IQR 7.0, 15.0), *p* < 0.001), worse function as measured by the CHAQ (median 0.8 (IQR 0.3, 1.5) vs 0.4 (IQR 0.0, 1.0), *p* < 0.001), and more pain (median 5.0 (IQR 2.0, 7.0) vs 4.0 (IQR 1.0, 6.0), *p* = 0.036) than those who started it > 3 months after diagnosis. Those who started ETN > 3 months after diagnosis were more likely to discontinue a non-biologic DMARD within 3 months of ETN initiation compared to those who started ETN ≤3 months after diagnosis (28.8% vs 17.1%, *p* = 0.005, data not shown). There was no statistically significant difference in the proportion of individuals with either sacroiliitis or enthesitis who started ETN ≤3 months after diagnosis compared to those who started it > 3 months (17.4 and 10.1% vs 12.9 and 5.3%, *p* = 0.30 and *p* = 0.16 respectively). In this more recent cohort, the median time from diagnosis to starting ETN was 4.3 months (IQR 1.3, 16.0), and from first non-biologic DMARD to ETN initiation was 3.2 months (IQR 0.9, 9.0).

Table 2 shows characteristics by ILAR categories for patients in the ETN Initiator Cohort. Patients whose subcategory of OligoJIA was not known (*n* = 22) were excluded from comparisons by ILAR category. As only a small proportion of the clinical cohort had a history of uveitis (< 1%, data not shown), this was not further analyzed. As expected, RF + pJIA patients had the highest active joint count (*p* < 0.001), highest cJADAS10 (*p* = 0.002), and worst functional status as measured by the CHAQ (*p* = 0.016) when compared to all other categories of JIA (2 group comparisons using Wilcoxon rank sum tests corrected for multiple comparisons). ERA patients were significantly more likely than other ILAR categories not to be taking any concurrent non-biologic DMARD with ETN (2 group comparison by χ^2^ corrected for multiple comparisons, *p* < 0.001), which is in keeping with the observation in the MTX assessment cohort that ERA patients were more likely than other JIA categories to start ETN without any prior MTX use. Overall, most individuals in the ETN Initiator Cohort who were treated with a non-biologic DMARD at ETN initiation continued this therapy for more than 3 months after starting ETN, though the proportion of patients doing so differed by ILAR category (*p* < 0.001).

**Table 2:** Characteristics of patients at start of ETN^a^ who had a clinical visit within 30 days, by JIA category^b^ (ETN Initiator Cohort, *n* = 443)

	PersistOligo^d^	ExtOligo^e^	RF + pJIA^f^	RF-pJIA^g^	ERA^h^	Psoriatici^i^	SJIA^j^	Undiff^k^
Number of patients^c^	53	30	64	192	57	33	3	11
Age at diagnosis in years, median (IQR)	5.0 (2.0, 10.0)	5.0 (3.0, 8.5)	13.0 (10.0, 15.0)	10.0 (5.0, 13.0)	11.0 (9.0, 14.0)	9.0 (2.0, 14.0)	6.0 (3.0, 17.0)	14.0 (11.0, 15.0)
Active^l^ enthesitis (%)^l^	3.8	10.0	6.3	7.8	56.1	24.2	0	18.2
Active sacroiliitis (%)	0	3.3	1.6	2.1	36.8	12.1	0	18.2
Active joint count, median (IQR)	1.5 (1.0, 2.0)	3.5 (1.0, 6.0)	7.5 (5.0, 16.5)	6.0 (3.0, 14.0)	3.0 (1.0, 9.0)	5.0 (2.0, 8.0)	2.0 (1.0, 20.0)	4.0 (3.0, 12.0)
Prior steroid injection (%)	67.9	73.3	15.6	22.4	17.5	18.2	0	27.3
Hx^m^ of 1 DMARD^n,o^ (%)	75.5	84.0	50.8	60.0	48.2	51.5	0	54.5
Any MTX^p^ (%)	75.5	80.0	50.8	58.9	44.6	45.5	0	27.3
Hx of > 1 DMARD (%)	5.7	12.0	3.3	3.8	7.1	6.1	0	0
1 biologic prior to ETN (%)	1.9	6.9	3.1	6.3	7.0	9.1	0	0
No concurrent DMARD with ETN (%)	24.5	26.9 (7/26)	9.7 (6/62)	16.0 (30/187)	42.9	30.3	66.7	36.4
DMARD for ≤3 months after ETN start (%)	41.5	38.5 (10/26)	24.6 (15/61)	21.5 (40/186)	16.1	24.2	33.3	18.2
DMARD for > 3 months after ETN start (%)	34.0	36.0 (9/25)	67.7 (42/62)	64.5 (120/186)	41.1	45.5	0	54.5
CHAQ^q^, Median (IQR)	0.1 (0.0, 0.6)	0.4 (0.0, 0.8)	1.1 (0.4, 1.5)	0.6 (0.1, 1.3)	0.5 (0.3, 0.9)	0.6 (0.3, 1.1)	0.5 (0.1, 0.9)	0.7 (0.3, 1.0)
cJADAS^r^ Median (IQR)	7.0 (4.5, 11.0)	11.0 (7.5, 15.0)	18.5 (12.0, 22.0)	15.0 (9.3, 20.0)	11.5 (7.8, 16.0)	13.0 (8.0, 17.0)	11.5 (11.5, 11.5)	11.0 (7.5, 14.0)
Physician Global Median (IQR)	3.0 (2.0, 4.0)	3.0 (2.0, 4.0)	5.0 (3.0, 7.5)	4.0 (2.8, 6.0)	3.0 (2.0, 5.0)	4.0 (2.0, 5.0)	5.0 (3.5, 6.5)	3.0 (2.0, 5.0)
Patient Global, Median (IQR)	2.5 (0.0, 5.0)	3.0 (1.0, 5.0)	5.0 (3.0, 7.0)	4.0 (2.0, 6.0)	4.0 (2.0, 6.0)	3.0 (2.0, 6.0)	6.5 (6.0, 7.0)	2.0 (1.0, 5.0)
Pain^s^, Median (IQR)	2.5 (0.0, 6.0)	4.5 (2.0, 6.0)	5.0 (3.0, 7.0)	4.0 (2.0, 6.0)	5.0 (3.0, 7.0)	4.0 (1.0, 5.0)	5.5 (4.0, 7.0)	5.0 (3.0, 6.0)

^a^*ETN* etanercept; ^b^ excludes oligoarticular JIA with unknown course; ^c^ percentages indicate percent of ILAR category, percentages were calculated for available n for each row, JIA category was determined at ETN initiation (prior to or up to 30 days after or closest visit to initiation);^d^*PersistOligo* persistent oligoarticular JIA, ^e^*ExtOligo* extended oligoarticular JIA, ^f^*RF + pJIA* polyarticular rheumatoid factor positive JIA, ^g^*RF–pJIA* polyarticular rheumatoid factor negative JIA, ^h^*ERA* enthesitis related arthritis, ^i^*Psoriatic* psoriatic JIA, ^j^*SJIA* systemic JIA, ^k^*Undiff* undifferentiated JIA, ^l^*active* clinically active, ^m^*Hx* history at any time > 1 month prior to starting ETN, ^n^*DMARD* non-biologic disease modifying anti-rheumatic drug; ^o^ medication data was not usable in certain cases, therefore the total of the three categories may add up to > 100%, denominators are provided for JIA categories in which this occurred, ^p^*MTX* methotrexate, ^q^*CHAQ* Childhood Health Assessment Questionnaire, ^r^*cJADAS10* clinical juvenile arthritis activity score (10 joints); ^s^ measured by a Likert scale from 0 (no pain) to 10 (worst pain)

Table 3 shows patients characteristics by pattern of MTX use in the ETN Initiator Cohort. There was no predominant treatment strategy for clinically active enthesitis. Patients with clinically active sacroiliitis were most likely to receive ETN alone (39.4%), followed by step-up therapy (24.2%), however the number of patients with sacroiliitis was small, precluding further statistical comparisons. Treatment approach varied with active joint count (*p* < 0.001). Patients initially treated with combination therapy had the highest median joint count, followed by those who received MTX add-on therapy, however the latter group was small and of uncertain significance. The median CHAQ, cJADAS10, physician global, patient/parent global, and pain score significantly differed among the 6 approaches.

**Table 3:** Clinical features and patterns of methotrexate (MTX) use at etanercept (ETN) initiation^a^ (ETN Initiator Cohort, *n* = 465)

Characteristic	Combination therapy^b^	Step-up therapy^c^	ETN proximate switchers^d^	ETN remote switchers^e^	MTX add-on^f^	ETN only	***p***-value^g^
Active^h^ enthesitis (*n* = 67), n (%)	13 (19.4)	15 (22.4)	11 (16.4)	6 (9.0)	3 (4.5)	19 (28.4)	0.073
Active sacroiliitis (*n* = 33), n (%)	4 (12.1)	8 (24.2)	5 (15.2)	2 (6.1)	1 (3.0)	13 (39.4)	
Active joint count, n	91	187	56	31	14	78	
Median (IQR)	11.0 (4.0, 20.0)	4.0 (2.0, 8.0)	2.0 (1.0, 8.0)	3.0 (1.0, 8.0)	9.0 (4.0, 17.0)	4.0 (1.0, 7.0)	<.001
CHAQ^i^, n	83	146	42	22	11	65	
Median (IQR)	0.9 (0.4, 1.5)	0.4 (0.1, 1.0)	0.6 (0.1, 1.1)	0.3 (0.0, 0.6)	0.8 (0.4, 2.0)	0.6 (0.0, 1.0)	<.001
cJADAS 10^j^, n	81	145	47	23	8	61	
Median (IQR)	19.0 (13.0, 22.0)	11.0 (7.5, 16.0)	10.5 (6.0, 16.0)	11.0 (6.0, 16.0)	18.8 (14.0, 23.0)	11.5 (8.0, 17.0)	<.001
Physician Global, n	89	175	50	29	13	74	
Median (IQR)	5.0 (3.5, 7.0)	3.0 (2.0, 5.0)	3.0 (2.0, 4.0)	3.0 (2.0, 4.0)	6.0 (3.0, 7.0)	4.0 (2.0, 6.0)	<.001
Patient Global, n	82	151	49	24	9	69	
Median (IQR)	5.0 (2.0, 6.0)	3.0 (1.0, 5.0)	4.0 (1.0, 5.0)	3.0 (1.5, 5.0)	6.0 (4.0, 6.0)	4.0 (2.0, 6.0)	0.022
Pain^k^, n	66	123	34	19	8	56	
Median (IQR)	5.0 (2.0, 7.0)	3.0 (2.0, 6.0)	4.5 (2.0, 7.0)	4.0 (1.0, 7.0)	6.5 (5.0, 9.0)	4.5 (2.0, 6.0)	0.014

^a^ Study visit must have been within 30 days prior to or after date of etanercept (ETN) initiation; ^b^ combination therapy = methotrexate (MTX) started concurrently with ETN; ^c^ step-up therapy = MTX started > 1 month prior to ETN and continued > 1 month after ETN; ^d^ proximate switchers = MTX started> 1 month prior to ETN and discontinued within 1 month prior to or after ETN; ^e^ ETN remote switchers = MTX discontinued > 1 month prior to start of ETN; ^f^ MTX add-on = MTX started > 1 month after starting ETN; ^g^*p*-values were calculated across all JIA categories using Pearson’s Chi Square or Fisher’s exact tests for categorical variables, and Wilcoxon rank sum, Kruskal-Wallis, or analysis of variance (ANOVA) for continuous variables depending on normality and the number of groups being compared; ^h^*active* clinically active, ^i^*CHAQ* Childhood Health Assessment Questionnaire, ^j^*cJADAS 10* clinical Juvenile Arthritis Disease Activity Score (10 joints); ^k^ Pain measured on a Likert scale from 0 (no pain) to 10 (worst pain)

**Fig. 1:**
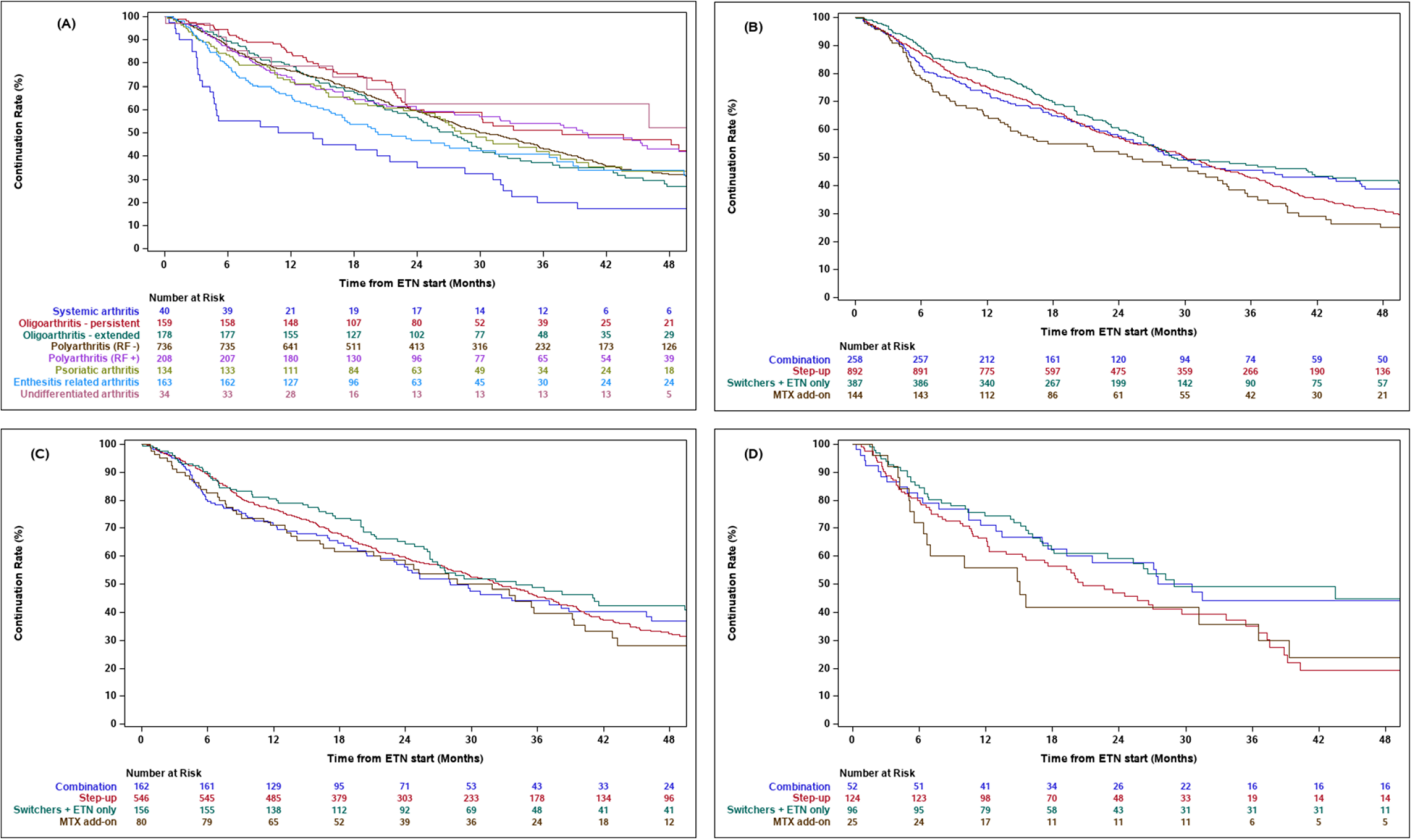
Kaplan-Meier curves showing Etanercept (ETN) persistence in the Methotrexate (MTX) Assessment Cohort (*n* = 1681); **a** by JIA category (Log Rank Test *p* < 0.001); **b** by pattern of MTX use at ETN initiation (Log Rank *p* = 0.002); **c** by pattern of MTX use at ETN initiation in polyarticular JIA (Log Rank *p* = 0.09); **d** by pattern of MTX use at ETN initiation in spondyloarthritis patients (enthesitis related arthritis and psoriatic JIA, Log Rank *p* = 0.03). Included patients had at least 1 study visit 6 months after starting ETN. Combination therapy = MTX started concurrently with ETN; step-up therapy = MTX started > 1 month prior to ETN and continued > 1 month after ETN initiation; switchers included proximate switchers (MTX started> 1 month prior to ETN and discontinued within 1 month) and remote switchers prior to or after ETN) and remote switchers (MTX discontinued > 1 month prior to start of ETN); MTX add-on = MTX started > 1 month after starting ETN

At 24 months overall ETN persistence was 66.3%, with lower persistence for SJIA (42.5%) and ERA (52.8%) than other JIA categories (Fig. 1a). At 36 and 48 and months overall persistence rates were 49.4 and 37.3% respectively. (Persistence rates over time are shown in Supplemental Table 1.) Fig. 1b shows ETN persistence by pattern of MTX use at ETN initiation. Persistence rates during the first 24 months were similar across all groups. Differences began to appear around 42 months, following which combination therapy and switchers demonstrated higher ETN persistence rates than patients in the step up the therapy group, and the MTX add-on group had the lowest level of ETN persistence. ETN persistence did not differ by MTX initiation pattern among RF+ and RF- pJIA patients (Fig. 1c). In contrast, ETN persistence among spondyloarthritis patients (ERA and PsJIA) varied by MTX initiation pattern. This can be seen in Fig. 1d beginning around 18 months after ETN initiation, with higher ETN persistence rates in the combination (68.9%) and switchers/ETN only (73.3%) patients compared to step-up (65.4%) and MTX add-on (51.1%) groups, a difference that remained visible over time. The MTX add-on population was very small beyond 30 months, potentially affecting the robustness of estimates for this group towards the end of the study period.

## Discussion

In this study, we describe contemporary patterns of ETN use and MTX use at ETN initiation in CARRA Registry JIA patients. Key findings include: (1) Patients with persistent oligoarticular JIA commonly switched from methotrexate to etanercept rather than stepping up therapy; (2) A substantial proportion of patients across JIA categories continued etanercept for 3 years or more; and (3) In spondyloarthritidies, etanercept persistence beyond 18 months varied with pattern of methotrexate use at etanercept initiation.

The proportion of females and ethnic distribution of patients were in keeping with expectations for JIA. The relatively high proportion of patients with polyarticular disease is expected, as the Registry initially preferentially enrolled patients with polyarticular course JIA and those treated with biologics. The proportions of RF-pJIA (42.6%) and RF + pJIA (11.9%) are similar to those reported in the British Society for Paediatric and Adolescent Rheumatology Etanercept Cohort Study (BSPAR-ETN) from 2010 to 2014 (39% RF-pJIA, 12% RF + pJIA) [15], but are higher than reported in some other registries that did not preferentially enrol a polyarticular phenotype [16]. Relatively few patients in the CARRA JIA Registry with SJIA were treated with ETN, likely because ETN is less effective in SJIA [17], is not FDA approved for SJIA, and anti-TNF agents have been replaced by FDA approved therapies targeting IL-1 or IL-6 in this category of JIA [18, 19]. The vast majority of patients with non-systemic JIA received ETN as their first biologic, which is in keeping with a number of other JIA registries, such as the Dutch National Arthritis and Biologicals in Children (ABC) registry (data from 1999 to 2010) [20], the German Biologika in der Kinderrheumatologie (BiKeR) registry, the Swedish JIA registry, and Pharmachild (data from 2010 to 2014) [16].

Since ETN was first approved for use in JIA, there has been a paradigm shift in the approach to treatment of inflammatory arthritis, with earlier use of biologic therapy particularly in the presence of highly active disease or risk factors for poor prognosis [10]. Not surprisingly, therefore, those who started ETN within 3 months of diagnosis in the CARRA Registry were more likely to have markers of more severe disease (higher joint count, worse cJADAS10, worse function, and more pain) than those treated with ETN later in their disease course. Overall, ETN was started relatively soon after diagnosis. In the larger cohort of all eligible patients, 75% of patients started ETN within 10.4 months after the first non-biologic DMARD, and within 21.8 months after diagnosis; while in the more recent ETN Initiator Cohort those numbers were even shorter (9 and 16 months respectively), likely reflecting increasing use of biologics over time and earlier aggressive therapy.

ACR JIA treatment guidelines for active polyarthritis recommend adding a biologic if an inadequate response is observed to MTX by 3 months, or if no or minimal response is observed in 6–8 weeks. These guidelines also recognize that in cases with high disease activity or risk factors for poor prognosis combining a non-biologic DMARD and a biologic at the start of therapy may be warranted [9, 10]. Additionally, CARRA consensus treatment plans (CTPs) for new onset polyarticular non-SJIA include three treatment strategies for starting biologics: step-up therapy in which a biologic is added to non-biologic DMARDs if needed after several months early combination therapy (biologic and non-biologic DMARD started together), and biologic monotherapy [21]. All three of these approaches were used by paediatric rheumatologists in the CARRA JIA Registry when starting ETN, and the predominance of step-up therapy is in keeping with ACR recommendations, with the caveat that we did not assess the proportion of patients who received a 3-month trial of MTX prior to treatment escalation or whether the time frame between medication changes were in keeping with the CTPs [9, 10]. A high proportion of patients received early combination therapy, also in keeping with 2019 ACR guidelines [10], and these patients had the highest median active joint counts overall. However, there was surprising variability in patterns of non-biologic DMARDs use prior to starting ETN. Approximately a third of patients were prescribed ETN without having been treated with an antecedent non-biologic DMARD. MTX was the most commonly prescribed non-biologic DMARD, but the route of administration was also quite variable, with subcutaneous administration most common, followed by both oral and subcutaneous routes, and slightly less commonly oral administration only.

Interestingly, patients with ERA were significantly more likely to report no concurrent non-biologic DMARD use (Table 1). Additionally, ERA patients had similar likelihoods of MTX step-up therapy and ETN monotherapy (Table 1). The patients in the MTX Assessment cohort did not all have clinical data collected at the time ETN was started, and therefore the role of sacroiliitis in the treatment choices for ERA patients could not be assessed in this cohort. Among patients in the smaller ETN Initiator Cohort, ETN monotherapy was the most common approach when sacroiliitis was present, though the number of patients with sacroiliitis was small (Table 3). The presence or absence of sacoiliitis could explain the findings in ERA patients in the larger cohort because sacroiliitis is most commonly seen in ERA, MTX is not recommended for sacroiliitis, and anti-TNF therapy is warranted if non-steroidal anti-inflammatories do not control sacroiliitis [9, 10].

Although the addition of ETN for treatment resistant persistent OligoJIA has been reported in the Dutch ABC Registry [22] and is in keeping with ACR treatment guidelines [10], its use for this category of JIA remains off-label. In our study 75.5% of persistent OligoJIA patients in the ETN Initiator Cohort had been treated with a non-biologic DMARD; however, only 34.0% of persistent OligoJIA continued treatment with a non-biologic DMARD 3 months or more after ETN initiation (Table 2). In the larger MTX Assessment Cohort, 15.1% of persistent OligoJIA were either proximate or remote switchers (who had discontinued MTX more than 1 month prior to starting ETN) (Table 1). Thus persistent OligoJIA patients in both of these cohorts were commonly switching to ETN rather than stepping up therapy and continuing combination treatment with a non-biologic DMARD longer term. Possible explanations for this include lack of efficacy or intolerance of MTX.

A substantial proportion of patients across all categories of JIA continued ETN for more than 2 years (49.4 and 37.3% at 3 and 4 years respectively). It is possible that a portion of this time represents medication tapering, which was not captured in our analysis. Alternatively, given the initial Registry enrolment criteria, the patients with longer follow-up may be biased towards more severe disease, and this high level of ETN persistence may change as patients enrolled after entry criteria broadening are followed over time. Interestingly, ETN persistence rates varied according to pattern of MTX use at ETN initiation. This difference was particularly striking in patients with spondyloarthritis (ERA and PsJIA). We were not able to assess whether disease characteristics varied with MTX use at ETN initiation because clinical data at ETN initiation was not available for many of the patients in the larger cohort.

Although the CARRA JIA Registry is a large observational cohort from which much can be learned, there are several limitations to this study. Some of our study findings lack clear explanation, but these results serve as steps toward further understanding the real-world use of ETN in the treatment of JIA. For the most part, CARRA Registry JIA patients are not an inception cohort, and rather represent a convenience sample of patients seen at academic paediatric rheumatology centers that are CARRA Registry sites. As such, there is a potential for selection bias limiting the generalizability of results to other geographic areas or healthcare systems. Also, as previously described in the Methods, prior to 2017 there was preferential enrollment of patients with specific JIA phenotypes and treatments. Additionally, several characteristics (e.g., enthesitis) are collected at study visits only, making it possible for patients to have clinical features that start and resolve between study visits. The physician assigned JIA category was used, which could have led to misclassification of JIA categories. This study was not able to assess trends over time prior to 2015, however longitudinal analyses will be possible in the future. Finally, assessing treatment outcomes of ETN therapy was beyond the scope of this descriptive study.

## Conclusions

This study is the first to describe contemporary patterns of ETN use among JIA patients in the CARRA Registry. There was large variability in the pattern of MTX use in this cohort. Treatment strategies were in keeping with current ACR guidelines and CARRA CTPs. As more patients enter the Registry close to time of diagnosis, further work will be needed to assess clinical criteria associated with specific patterns of MTX use when staring ETN in patients with spondyloarthritis, and whether these patterns affect outcomes.

## Supplementary Information

**Additional file 1: Table S1**. Etanercept (ETN) persistence rates in the Methotrexate (MTX) Assessment Cohort^a^ (*n* = 1681).

## Data Availability

The data underlying this article were provided by the Childhood Arthritis and Registry Research Alliance (CARRA) by permission. Data may be shared on reasonable request to CARRA (carragroup.org).

## Abbreviations

ABC: Dutch National Arthritis and Biologicals in Children
ACR: American College of Rheumatology
ANOVA: Analysis of variance
BiKeR: Biologika in der Kinderrheumatologie
BSPAR-ETN: Paediatric and Adolescent Rheumatology Etanercept Cohort Study
CARRA: Childhood Arthritis and Rheumatology Research Alliance
CHAQ: Childhood Heath Assessment Questionnaire
cJADAS10: Clinical Juvenile Arthritis Disease Activity Score 10 joint
CTP: Consensus treatment plan
DMARD: Disease modifying anti-rheumatic drug
ETN: Etanercept
ERA: Enthesitis related arthritis
FDA: Food and Drug Administration
ILAR: International League Against Rheumatism
JIA: Juvenile idiopathic arthritis
MTX: Methotrexate
OligoJIA: Oligoarticular juvenile idiopathic arthritis
pJIA: Polyarticular juvenile idiopathic arthritis
PROMIS: Patient-Reported Outcome Measurement Information System
PsJIA: Psoriatic arthritis
IQR: Interquartile range
RF: Rheumatoid factor
SJIA: Systemic juvenile idiopathic arthritis
TNF: Tumor necrosis factor
