# Supplementary material for: Patterns of etanercept use in juvenile idiopathic arthritis in the Childhood Arthritis and Rheumatology Research Alliance Registry
Source: Pediatr Rheumatol Online J. 2021 Aug 21;19:131. doi: 10.1186/s12969-021-00625-y (PMC8380401; doi:10.1186/s12969-021-00625-y)
Supplement: Supplementary file 1 — Additional file 1: Table S1. Etanercept (ETN) persistence rates in the Methotrexate (MTX) Assessment Cohorta (n = 1681). [file 12969_2021_625_MOESM1_ESM.docx]

|  | **Month 12** | **Month 18** | **Month 24** | **Month 36** | **Month 48** |
| --- | --- | --- | --- | --- | --- |
| All JIA^b^ categories (%) | 86.1 | 75.1 | 66.3 | 49.4 | 37.3 |
| JIA category |  |  |  |  |  |
| Systemic arthritis | 52.5 | 47.5 | 42.5 | 30.0 | 17.1 |
| Oligoarthritis – persistent | 93.1 | 83.8 | 74.4 | 57.2 | 47.1 |
| Oligoarthritis – extended | 88.8 | 78.2 | 67.4 | 42.5 | 32.7 |
| Polyarthritis (RF -)^c^ | 87.1 | 76.4 | 68.7 | 49.8 | 35.4 |
| Polyarthritis (RF +) | 86.5 | 73.5 | 63.7 | 56.0 | 46.7 |
| Psoriatic arthritis | 82.8 | 71.0 | 62.6 | 46.8 | 33.4 |
| Enthesitis related arthritis | 77.9 | 65.7 | 52.8 | 40.9 | 39.3 |
| Undifferentiated arthritis | 82.4 | 74.0 | 68.7 | 68.7 | 52.1 |
| ETN persistence by MTX initiation pattern - all patients |  |  |  |  |  |
| Combination^d^ | 82.2 | 72.4 | 64.6 | 48.8 | 42.3 |
| Step-up^e^ | 86.9 | 75.1 | 66.9 | 49.8 | 34.7 |
| Switchers^f^ + ETN only | 88.4 | 80.6 | 69.4 | 48.6 | 42.6 |
| MTX add-on^g^ | 77.8 | 64.1 | 54.0 | 45.2 | 27.7 |
| ETN persistence by MTX initiation pattern – polys^h^ |  |  |  |  |  |
| Combination | 79.6 | 70.4 | 63.8 | 46.4 | 38.5 |
| Step-up | 88.8 | 76.7 | 67.9 | 52.1 | 36.8 |
| Switchers + ETN only | 88.5 | 79.8 | 72.8 | 50.9 | 47.5 |
| MTX add-on | 81.3 | 69.7 | 60.0 | 48.1 | 30.7 |
| ETN persistence by MTX initiation pattern – spondylo^i^ |  |  |  |  |  |
| Combination | 78.9 | 68.9 | 60.1 | 47.2 | 47.2 |
| Step-up | 79.0 | 65.4 | 55.2 | 37.3 | 32.7 |
| Switchers + ETN only | 83.3 | 73.3 | 60.9 | 57.4 | 44.9 |
| MTX add-on | 68.0 | 51.1 | 51.1 | 35.8 | 29.9 |

^a^in patients with at least 1 study visit 6 months after starting etanercept; ); ^b^JIA=juvenile idiopathic arthritis; ^c^RF=rheumatoid factor; ^d^Combination therapy=methotrexate (MTX) started concurrently with ETN; ^e^step-up therapy = MTX started >1 month prior to ETN and continued >1 month after ETN; ^f^switchers include proximate switchers (MTX started>1 month prior to ETN and discontinued within 1 month prior to or after ETN) and remot switchers (MTX discontinued >1 month prior to start of ETN; ^g^MTX add-on=MTX started >1 month after starting ETN ^h^polys=RF negative and RF positive polyarticular JIA; ^i^Spondylo=enthesitis related arthritis and psoriatic arthritis categories of JIA
